# Causes of Death in Childhood Acute Lymphoblastic Leukemia: A Single-Center Experience

**DOI:** 10.3390/medicina61071193

**Published:** 2025-06-30

**Authors:** Matej Jelić, Maja Pavlović, Lucija Mucavac, Sara Dejanović Bekić, Zrinko Šalek, Toni Matić, Daniel Turudić, Luka Lovrenčić, Jelena Roganović, Ernest Bilić

**Affiliations:** 1 Division of Hematology and Oncology, Department of Pediatrics, University Hospital Center Zagreb, Kispaticeva 12, 10000 Zagreb, Croatia; matejjelic1@gmail.com (M.J.); drmaja.pavlovic@gmail.com (M.P.); dejanovic.sara@gmail.com (S.D.B.); zrinko.salek@gmail.com (Z.Š.); madtmatic@yahoo.com (T.M.); danielturudic@gmail.com (D.T.); lovrencic.luka@outlook.com (L.L.); ernest.bilic@kbc-zagreb.hr (E.B.); 2 School of Medicine, University of Zagreb, Salata 3, 10000 Zagreb, Croatia; 3 Children’s Hospital Zagreb, 16 Vjekoslava Klaica 16, 10000 Zagreb, Croatia; 4 Faculty of Biotechnology and Drug Development, University of Rijeka, Radmile Matejcic 2, 51000 Rijeka, Croatia

**Keywords:** leukemia, children, death, prognostic factors, relapse, infection

## Abstract

Acute lymphoblastic leukemia (ALL) is the most common malignancy in children. Good overall survival rates of about 90% are the result of improvements in risk stratification and risk-adapted therapy, intensive chemotherapy regimens, hematopoietic stem cell transplantation, and better supportive care. *Background and Objectives*: The aim of this study is to review the epidemiology, prognostic factors, and causes of death in pediatric ALL patients treated at a tertiary care center, and to identify risk factors influencing clinical outcomes. *Materials and Methods*: A retrospective study was conducted at the Department of Pediatric Hematology and Oncology, University Hospital Centre Zagreb, including 302 children (0–18 years) diagnosed with ALL between January 2001 and December 2015. *Results*: Two hundred fifty-one children survived (5-year overall survival 83%). Relapse occurred in 13.6% of patients. Relapse rates were higher for B-cell precursor (Bcp)-ALL than for T-cell ALL (14.3% vs. 10.4%), and no patient with relapsed T-cell ALL survived. The main causes of death were refractory/relapsed disease (43% of patients), followed by infections (35%) and GVHD (8%). The most frequent causes of infectious death were *Pseudomonas aeruginosa* and *Aspergillus fumigatus*. The most critical treatment periods were the induction and reinduction phases, especially the de-escalation of corticosteroids. The time of relapse and risk group were independent factors in predicting the outcome. *Conclusions*: Relapse and infections were the leading causes of death in children with ALL, with the highest mortality observed during induction and reinduction phases. Survival was significantly influenced by relapse timing and risk group, with no survivors among relapsed T-ALL patients.

## 1. Introduction

Acute lymphoblastic leukemia (ALL) is the most common cancer in children, making up about one-fourth of all pediatric cancers [1]. The survival rate of pediatric ALL has improved significantly with risk stratification based on biologic features of leukemic cells and response to the treatment, therapy modifications based on patients’ pharmacodynamics and pharmacogenomics, and better supportive care [2]. These advances have improved the survival rate from less than 10% in the 1960s to about 90% nowadays [3]. The CONCORD study reported pooled age-standardized net survival in the US of 95% at 1 year and 86% at 5 years for children diagnosed in 2001–2003, increasing to 96% and 88%, respectively, for 2004–2009 [4]. A large multicenter trial in 13 resource-limited countries (ALL IC-BFM 2009) showed 5-year EFS and OS rates of 75.2% ± 0.5% and 82.6% ± 0.5%, aligning with the results from the previous ALL IC-BFM 2002 trial [5]. Similarly, Children’s Oncology Group (COG) data showed an increase in 5-year survival from 83.7% (1990–1994) to 90.4% (2000–2005), with the exception of infants ≤ 1 year [6].

When looking at treatment failure and mortality, two important comparisons can be made. First, there are clear differences between high- and low-income countries [7]. According to studies [8,9,10,11,12,13], relapse is a common cause of treatment failure in high-income countries (8–19%). Despite advances, post-relapse survival remains suboptimal, though gradual improvements have been observed over time. A 2018 COG (Children’s Oncology Group) study reported 3-year overall survival (OS) rates ranging from 29.7% to 38.1%, depending on the site of relapse [14]. In a 2022 cohort, the 5-year OS was 31.6%, showing little change compared to earlier data [14]. However, recent studies from 2024 suggest improving outcomes. Wang et al. reported the 5-year OS of 37  ±  4% and an event-free survival (EFS) of 26  ±  4% among 136 relapsed children [15]. Rheingold et al. showed even better results, with the 5-year OS of 48.9% overall: 52.5% for B-ALL, 35.5% for T-ALL, and 21.5% for infant ALL [16]. While differences remain across studies, these data reflect a positive trend in post-relapse survival in pediatric ALL [17]. Conversely, in low-income countries, infections are the leading cause of death in children with ALL (43.2%), followed by relapse (27%) [18]. Managing infections is particularly challenging due to poverty-related factors, malnutrition, and limited access to trained staff, diagnostics, and infrastructure [19]. Treatment-related mortality shows a clear inverse relationship with income level, reaching 14.2% in low-income countries, 9.2% in lower-middle-income countries, and 4.5% in upper-middle-income countries [20]. Other causes, such as treatment abandonment (9.5%) and bleeding (8.1%), are less common but still relevant [18]. Given the ongoing treatment-related mortality, especially in the early phase of therapy, there is a strong need for safer and more effective treatments. Conventional chemotherapy has reached its limits due to toxicity, particularly in children. Novel immunotherapies—such as blinatumomab, inotuzumab ozogamicin, and CAR T-cell therapy—offer promising alternatives by reducing toxicity while maintaining high efficacy [16,17]. The aim of this study is to review the epidemiology, prognostic factors, and causes of death in pediatric ALL patients treated at a tertiary care center, and to identify risk factors influencing clinical outcomes.

## 2. Materials and Methods

### 2.1. Study Design

A retrospective study was carried out at the Division of Hematology and Oncology, Department of Pediatrics, University Hospital Centre Zagreb, during the time period from 1 January 2001 to 31 December 2015. The Division is the Croatian Referral Center for Pediatric Hematology and Oncology, treating approximately 70% of all children diagnosed with ALL nationwide.

### 2.2. Participants and Data Collection

The study included 302 children aged 0–18 years who were newly diagnosed with ALL. Exclusion criteria were previous treatment and secondary ALL. Epidemiological and clinical data were obtained from patient records. Demographic information included gender, date of birth, age at diagnosis, and therapy start date. Other collected data included ALL immunophenotype, risk group, time and site of relapse, treatment protocol, date of last follow-up/date of death, and causes of death. Bone marrow cytogenetic data before 2010 were incomplete and not available in the hospital database; therefore, cytogenetic data were excluded from the analysis. Minimal residual disease (MRD) monitoring and molecular genetics were introduced in 2009, and since many patients were treated prior to this, MRD data were also incomplete and excluded from statistical analysis. The majority of the patients (96%) had initially been treated according to ALL-BFM 95, ALL-IC-BFM 2002, and ALL-IC-BFM 2009 protocols and classified into risk groups (standard risk—SR, intermediate risk—IR, and high risk—HR), while infants had been treated according to the Interfant protocol (Interfant 99, Interfant 06). Philadelphia chromosome–positive (Ph+) ALL-positive patients were treated according to EsPhALL2004/EsPhALL2010 Protocols, which involved hematopoietic stem cell transplantation (HSCT).

Patients were stratified according to the time of relapse as follows: very early relapse occurring less than 18 months from diagnosis, early relapse between 18 and 30 months from diagnosis, and late relapse occurring more than 30 months after diagnosis.

### 2.3. Statistical Analysis

Collected data were incorporated into a previously constructed database in Microsoft Excel 2010 (Microsoft Corporation, Redmond, WA, USA) and processed using Stata/MP version 13 (StataCorp LLC, College Station, TX, USA). Descriptive statistics, including frequencies, percentages, and medians, were calculated using the Excel Data Analysis Toolpak (Microsoft Corporation, Redmond, WA, USA). The results were interpreted at a significance level of *p* < 0.05. A Kaplan–Meier curve was used to analyze the survival rates. OS was defined as the time from diagnosis to death from any cause, and patients who were alive at the last follow-up were censored. Event-free survival (EFS) was defined as the time from diagnosis to the first event, including disease progression, relapse, second malignancy, or death from any cause. Patients without an event were censored at the time of last follow-up. Univariate and multivariate analyses (Efron method) by Cox proportional hazard model were performed with 95% confidence intervals (CIs) in order to identify statistically significant prognostic factors of survival.

## 3. Results

### 3.1. Patient Characteristics

The median age of patients was 5 years (range 9 months to 17 years). Female patients had a lower median age at diagnosis (4 years) compared to males (5 years). Five-year EFS was 80%, while 5-year OS was 83%. Patients who experienced relapse were included in the original cohort at the time of initial diagnosis. Characteristics of patients with ALL and differences in epidemiology between newly diagnosed and relapsed patients are shown in Table 1.

### 3.2. Relapsed Patient Characteristics

Relapse occurred in 41 patients (13.6%), with no statistically significant difference in relapse rate according to gender, although girls had better OS than boys (86% vs. 81%). Characteristics of ALL patients who experienced relapse are shown in Table 2.

### 3.3. Treatment Outcomes and Survival Rates

The relapse rate was higher in B-ALL (14.5%) than in T-ALL (10%). Five-year OS of patients without relapse (251 patients) was 93%, while 5-year OS of patients who experienced relapse was 22%; there was a statistically significant difference between these groups (Table 3). 

**Figure 1:**
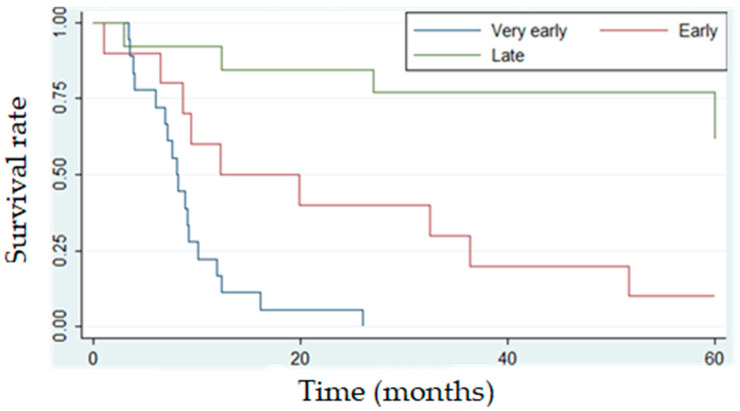
The 5-year OS of relapsed patients according to the time of relapse (Kaplan–Meier curve).

The median time from diagnosis to relapse was 20 months (for SR patients, 52 months; for IR patients, 12 months; and for HR patients, 8 months), while the median post-relapse survival time was 9 months. Figure 1 shows the 5-year OS of relapsed patients according to the time of relapse.

### 3.4. Mortality and Causes of Death

We recorded 51 total deaths with a cumulative incidence of 16.9%. Notably, 19 patients (37%) died without relapse, and 32 patients died (63%) after relapse. The main cause of death was refractory/relapsed disease (43%) followed by infections (35%), graft-versus-host disease (GVHD, 8%), hemorrhage (6%), liver failure (4%), discontinued treatment (2%), and unknown cause (2% of patients). The most critical periods in non-relapsed patients were the induction (eight patients, 42%) and reinduction phases of the treatment (nine patients, 48%), while only one patient died during intensification and one patient after HSCT.

**Figure 2:**
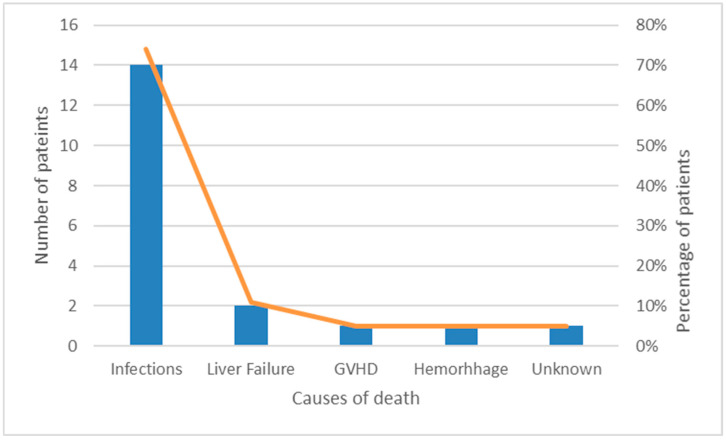
This figure presents the distribution of causes of death among non-relapsed patients, highlighting infections as the leading cause (14 patients, approximately 75%). Other causes, including liver failure (11%), GVHD (5%), and hemorrhage (5%), were less frequently observed.

**Figure 3:**
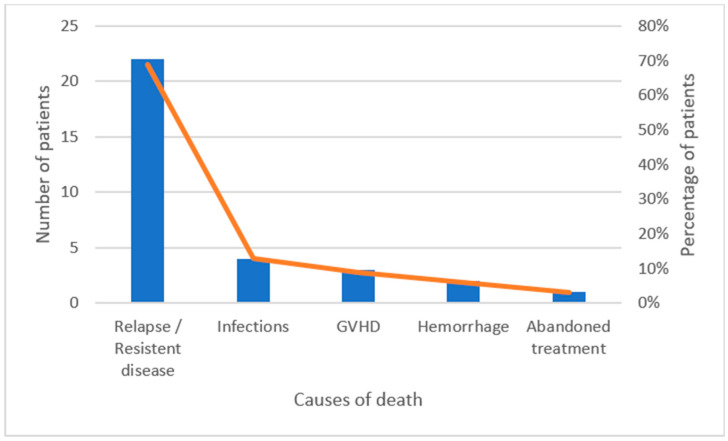
This figure illustrates the distribution of causes of death among relapsed patients, with relapse or resistant disease being the predominant cause (22 patients, approximately 70%). Other causes, such as infections, GVHD, hemorrhage, and abandoned treatment, account for progressively fewer deaths, representing 13%, 9%, 6%, and 3% of patients, respectively. Regarding infections, the most common isolated microorganisms were *Pseudomonas aeruginosa* (7 patients—5 non-relapsed and 2 relapsed), *Aspergillus fumigatus* (6 patients—5 non-relapsed and 1 relapsed), and *Fusarium spp.* (2 patients—1 non-relapsed and 1 relapsed).

Causes of death for non-relapsed and for relapsed patients are shown in Figure 2 and Figure 3.

The risks of death are shown in Table 3 and Table 4.

### 3.5. Treatment Protocols

We also analyzed the number of patients treated according to each protocol. The results are presented in Table 5. If patients treated according to Interfant and EsPhALL protocols were excluded, the 5-year OS was 84.2%.

## 4. Discussion

The five-year OS of children with ALL in our study was 83%. In larger studies, Jeha (St Jude Total Therapy XVI protocol) [21] and Pieters (DCOG 10) [22] reported 5-year OS rates of 94.1% and 92%, respectively. On the other side, the 10-year EFS in the study of Keizo [23] was 77% for patients recruited from 1997 to 2002 without a significant difference between risk groups—EFS rates for SR, IR, HR, and very HR groups were 79.3%, 72.5%, 71.7%, and 66.3%, respectively. Similar results were reported by Hao [18], with a 10-year OS of 69%, confirming that survival rates are related to the resources of the country. Notably, our study, unlike several other studies, includes patients without the exclusion of HR cases, such as Ph + ALL or infants.

Long-term survival of infants remains unsatisfactory despite many efforts for improvement with intensified chemotherapy and immunotherapy. Hossain et al. [24] reported the lowest survival rate (50%) in infants, compared to age groups 1–4, 5–9, 10–14, and 15–19 years (82%, 75%, 57%, and 32%, respectively). The survival rate is also inversely proportional to age in our cohort, except for infants, but in our study, multivariate regression analysis revealed no statistical significance. It should be mentioned that univariate regression analysis showed age groups as significant predictors of outcome, but this was not documented in multivariate analysis. Relapse rates in our patients were highest in infants (50%) and lowest in children 1–4 years (10%).

Immunophenotype is an important component in the diagnostic evaluation of patients with ALL. Many studies [17,18] reported a higher relative risk of death in patients with T-cell ALL. Our results closely match for B-cell and T-cell ALL (survival rates 83.3% and 82%, respectively). We observed higher relapse rates for B-cell ALL than for T-cell ALL (14.3% and 10%, respectively), but there were no survivors in the cohort of patients with T-cell ALL who experienced a relapse. Pro-B ALL remains infrequent but with the worst outcome. In the study of Rheingold and colleagues [25], relapse rates were similar for B- and T-cell ALL (12% and 11%), and the prognosis was better for patients with relapsed B-ALL (52 ± 1%) than for T-ALL (33 ± 3%) and infant ALL (19 ± 4%), with greater variability in OS by the site in T-ALL vs. B-ALL. In multivariable analysis, their results were equal to ours, showing that a shorter time to relapse was associated with the worse outcome; in addition to the site of relapse, age < 1 or >10 years at diagnosis, initial WBC > 100 × 10^12^/L, and T-cell phenotype were identified as statistically significant unfavorable prognostic factors. The small sample size in our study makes the conclusion about variability in OS by the site of relapse unreliable.

Risk-adapted therapy is one of the main contributors to the significant improvement in survival in children with ALL. The OS for SR patients in our study was 96%, with only 6% of relapsed patients, which is comparable to the results of Maloney et al. [26], with the 6-year OS for children with SR ALL enrolled in AALL0331 exceeding 95%.

As reported in other studies [12,27], treatment-related mortality, mainly as a result of infections, primarily occurred in induction protocols. In our study, deaths were slightly more reported in the reinduction protocol (nine deaths in reinduction and eight deaths in induction). Furthermore, we want to emphasize the tapering of corticosteroids as the critical period for susceptibility to infection. Inaba et al. [28] noted that poor neutrophil surge after dexamethasone pulses during continuation, which can reflect the poor bone marrow reserve, was associated with infections.

Nakagawa et al. [29] reported that fatal infections mostly occurred during the third week of induction therapy. They suggested close monitoring, stringent infection control, and immediate administration of appropriate antibiotics through hospitalization as an approach to reducing the rate of infection-related induction deaths. Our patients were hospitalized throughout the entire induction phase, and during reinduction, they were discharged between therapies if their general condition and laboratory findings were acceptable. We evaluated patients regularly in an outpatient setting, and they were admitted at the slightest suspicion of infection or deterioration. Microbiological specimens (stool, urine, oropharynx, nasopharynx, armpit, and groin) were collected routinely at least once a week.

The most common isolated microorganisms as the cause of sepsis and death in our study were *Pseudomonas aeruginosa*, followed by *Aspergillus fumigatus* and *Fusarium* spp. Also, Nakagawa [29] reported *Pseudomonas aeruginosa* as the most common cause of sepsis and death (32%), followed by *Bacillus* spp. (27%), while other microorganisms (*Enterobacter cloacae*, *Candida albicans*, *Aspergillus*, *Candida tropicalis*, and *Chickenpox*) were rare causes of death. Ruijters and colleagues [30] reported that the most common fungal cause was *Candida*, followed by *Aspergillus*.

Due to the growing incidence of invasive fungal infections (IFIs), many studies [31,32,33,34] focused on preventive strategies and reported the importance of environmental factors. Building constructions, tobacco, pets, potted plants, gardening, and rooms without HEPA filtration might increase the risk of IFIs. Short-term laminar airflow use may reduce the risk of aspergillosis, but its long-term use is inadequate.

In this study, relapse occurred in 51 patients (13.6%) which is higher than in UKALL2003 trial (relapse risk 8.8%) [4] and DFCI 05-001 trial (relapse risk 8.9%) but slightly lower in comparison with the study of Tuong et al. [35] (relapse risk 16.7%). Most of our patients (43.9%) had a very early relapse with a dismal prognosis, while early and late relapses were associated with better yet still poor prognosis (10% and 62%, respectively). The time of relapse was an independent unfavorable prognostic factor, which is comparable with an Egyptian study [36] reporting that ‘very early’ relapses had significantly lower 5-year OS (15.8%) compared to ‘early’ and ‘late’ relapses (46.2% and 66.7%, respectively), without significance in multivariate Cox regression analysis. Kelly et al. [37] reported that EFS was significantly higher in patients with a longer duration of the first complete remission, regardless of whether the threshold was set at 24 months (*p* < 0.05) or 36 months (*p* < 0.05), which is in agreement with our results. Furthermore, their study showed no significant difference between disease-free survival (DFS) at 8 years for patients treated with chemotherapy alone (45.3% ± 11.2%) and chemotherapy followed by SCT (50% ± 9.1%).

Regardless of the better results reported in some studies after relapse [17], extremely aggressive treatment for ALL relapse is often associated with substantial late sequelae. Therefore, improved strategies to prevent relapse are needed for the subset of HR patients. Pui et al. [11] reported that patients with NCI HR B-ALL or T-ALL had an inferior outcome, even with undetectable minimal residual disease (MRD) on day 46, with the cumulative risk of relapse of 12.7% and 15.5%, respectively.

The analysis of OS and relapse rates according to treatment protocols in our cohort reveals subtle differences when compared with published data. For patients treated with the ALL-BFM 95 protocol, our observed 5-year OS was 81.5%, with a relapse rate of 16.7%. This compares reasonably with the original trial’s 6-year EFS of 79.6% [38]. While the OS is slightly higher in our data, relapse rates remain moderate. The ALL IC-BFM 2002 protocol showed the highest 5-year OS in our population (85.8%), consistent with published survival data of 82% for the entire cohort and 83% for IR patients [9]. Similarly, our results with ALL IC-BFM 2009 (83.5% OS, 9.7% relapse rate) align well with published data, which reported a 5-year OS of 82.6% and EFS of 75.2%. Both studies highlight strong outcomes in the IR group, which matches the largest portion of our cohort [5].

The EsPhALL2004/2010 subgroup in our cohort included only three patients, limiting statistical interpretation; however, their OS of 66.7% and relapse rate of 33.3% were somewhat lower than expected given the known benefit of TKI-based therapy in Ph+ ALL. The EsPhALL2004 trial showed a 10% improvement in disease-free survival with short, discontinuous imatinib after induction compared to chemotherapy and HSCT alone. This led to EsPhALL2010, which introduced early and continuous imatinib from day 15 of induction. Although fewer patients underwent HSCT in EsPhALL2010 (38%), 5-year OS remained favorable at 71.8%, with EFS of 57.0%, supporting the value of prolonged TKI exposure despite increased toxicity during intensive treatment phases [39,40].

In our cohort, the Interfant 99/06 subgroup showed poor outcomes, with a 5-year OS of 50.0% and relapse rate of 50.0%, aligning with historical data. The Interfant-06 trial reported a 6-year OS of 58.2% and EFS of 46.1%, with even lower EFS (20.9%) in high-risk patients. However, recent data suggest promising improvements with blinatumomab added to Interfant-06 chemotherapy, yielding a 2-year disease-free survival of 81.6% and OS of 93.3%, significantly better than historical controls. These findings highlight the potential of immunotherapy to improve outcomes in infants with KMT2A-rearranged ALL [41].

Our goal is to reach better results comparable to highly developed centers using standardized treatment protocols for relapse and better supportive care. Further studies are needed to focus on molecular therapies and cellular immunotherapy to achieve better survival, reduce treatment-related mortality, and improve the quality of life in childhood cancer survivors.

Our study has some limitations. It is a retrospective, single-center study, which may cause some bias and limit how well the results apply to other places. Bone marrow cytogenetics data before 2010 and MRD data before 2009 were incomplete, so we could not include them in the analysis. Additionally, during the study period, treatment protocols and supportive care changed, which could affect the results and make comparisons difficult.

## 5. Conclusions

This study demonstrated a 5-year OS rate of 83% and a relapse rate of 13.6% in children with ALL. Infants and patients with very early relapse had the poorest prognosis. If patients with Ph+ ALL and infants are excluded, the 5-year OS increases to 84.2%. Treatment-related mortality was primarily driven by infections, with most deaths in non-relapsed patients occurring during induction or reinduction. Overall, 51 patients (16.9%) died, mostly due to relapsed/refractory disease (43%) and infections (35%). Notably, 5-year OS was 93% in patients without relapse, compared to 22% in those who relapsed. Despite limited resources, our results are comparable to those reported by more developed centers, highlighting the effectiveness of standardized treatment protocols and comprehensive care delivery.

## Figures and Tables

**Table 1:** Characteristics of patients with acute lymphoblastic leukemia.

	Number of Patients (%)	5-Year EFS (%)	5-Year OS (%)	Number of Relapsed Patients	Percentage of Relapsed Patients
					In Relation to Overall Number at the Diagnosis (%)
Patients	302 (100%)	80%	83%	41	14%
Gender					
Male	178 (59%)	77%	81%	30	17%
Female	124 (41%)	85%	86%	11	9%
Age groups					
Infants	8 (3%)	50%	50%	4	50%
1–4 y.	165 (55%)	85%	88%	16	10%
5–9 y.	66 (22%)	77%	83%	11	17%
10–17 y.	63 (21%)	73%	73%	10	16%
Immunophenotype					
common	181 (60%)	82%	86%	22	12%
pre B	63 (21%)	73%	78%	12	19%
pro B	5 (1%)	60%	60%	2	40%
T	53 (18%)	83%	83%	5	10%
Risk groups					
SR	69 (23%)	87%	96%	4	6%
IR	154 (51%)	85%	89%	16	10%
HR	79 (26%)	53%	63%	21	27%

Abbreviations: y.—years, SR—standard risk, IR—intermediate risk, HR—high risk, EFS—event-free survival, OS—overall survival.

**Table 2:** Characteristics of patients with relapsed acute lymphoblastic leukemia.

	Number	Percentage	Survival	5-Year OS (%)
Patients	41	100%	9	22%
GENDER				
Male	30	73%	7	23%
Female	11	27%	2	18%
AGE GROUPS				
Infants (0 to <12 months)	4	10%	0	0%
1–4 y.	16	39%	5	31%
5–9 y.	11	27%	4	36%
10–17 y.	10	24%	0	0%
IMMUNOPHENOTYPE				
common	22	54%	6	27%
pre B	12	29%	3	25%
pro B	2	5%	0	0%
T	5	12%	0	0%
RISK GROUPS				
SR	4	10%	1	25%
IR	16	39%	7	44%
HR	21	51%	1	5%
TIME OF RELAPSE				
Very early	18	44%	0	0%
Early	10	24%	1	10%
Late	13	32%	8	62%
SITE OF RELAPSE				
Bone marrow	27	66%	6	22%
Isolated CNS	1	2%	1	100%
Bone marrow + CNS	13	32%	2	15%
TREATMENT				
Chemotherapy	22	54%	3	14%
Chemotherapy + HSCT	19	46%	6	32%
SITE OF RELAPSE				
Bone marrow	27	66%	6	22%
Isolated CNS	1	2%	1	100%
Bone marrow + CNS	13	32%	2	15%
TREATMENT				
Chemotherapy	22	54%	3	14%
Chemotherapy + HSCT	19	46%	6	32%

Abbreviations: CNS—central nervous system, HSCT—hematopoietic stem cell transplantation.

**Table 3:** Hazard risk of mortality associated with prognostic factors in pediatric ALL patients.

Analysis	Univariate HR	*p*	Multivariate HR	*p*
INITIAL DIAGNOSIS				
Relapse				
No	1.00	Reference	1.00	Reference
Yes	15.78 (8.76–28.43)	<0.05	13.24 (6.92–25.37)	<0.05
Sex				
Male	1.00	Reference	1.00	Reference
Female	0.66 (0.37–1.2)	0.17	1.02 (0.54–1.91)	0.96
Age groups				
Infants	1.00	Reference	1.00	Reference
1–4 y.	0.19 (0.06–0.55)	0.002	1.07 (0.33–3.54)	0.9
5–9 y.	0.24 (0.08–0.77)	0.016	0.66 (0.19–2.3)	0.52
10–17 y.	0.43 (0.14–1.28)	0.128	1.03 (0.33–3.27)	0.95
Immunophenotype				
common	1.00	Reference	1.00	Reference
pre B	1.72 (0.89–3.31)	0.104	1.41 (0.72–2.77)	0.32
pro B	3.6 (0.85–15.22)	0.082	1.48 (0.32–6.73)	0.61
T ALL	1.3 (0.61–2.78)	0.502	1.75 (0.77–3.98)	0.18
Risk group				
SR	1.00	Reference	1.00	Reference
IR	2.51 (0.73–8.64)	0.142	2.18 (0.61–7.75)	0.23
HR	11.76 (3.59–38.5)	<0.05	6.85 (1.87–24.9)	<0.05

Abbreviations: y.—years, SR—standard risk, IR—intermediate risk, HR—high risk.

**Table 4:** Hazard risk of mortality associated with prognostic factors in relapsed pediatric ALL patients.

Analysis	Univariate HR	*p*	Multivariate HR	*p*
RELAPSED PATIENTS				
Sex				
male	1.00	Reference	1.00	Reference
female	1.02 (0.47–2.2)	0.95	1.06 (0.44–2.57)	0.897
Age groups				
infants	1.00	Reference	1.00	Reference
1–4 y.	0.14 (0.04–0.5)	<0.05	0.36 (0.05–2.78)	0.33
5–9 y.	0.14 (0.03–0.52)	<0.05	0.81 (0.14–4.69)	0.81
10–17 y.	0.29 (0.08–1.03)	0.055	0.72 (0.14–3.59)	0.69
Immunophenotype				
common	1.00	Reference	1.00	Reference
pre B	1.96 (0.87–4.4)	0.102	2.16 (0.66–7.1)	0.21
pro B	23.56 (3.74–148.2)	<0.05	7.17 (0.76–67.76)	0.086
T ALL	9.1 (2.77–29.9)	<0.05	2.87 (0.49–16.84)	0.24
Risk group				
Standard risk	1.00	Reference	1.00	Reference
Intermediate risk	0.95 (0.26–3.5)	0.94	3.08 (0.44–21.69)	0.26
High risk	3.57 (1.04–12.27)	0.043	14.18 (1.2–167.4)	0.035
Time of relapse				
very early	1.00	Reference	1.00	Reference
early	0.1 (0.03–0.32)	<0.05	0.22 (0.03–1.55)	0.129
late	0.02 (0.004–0.08)	<0.05	0.008 (0.001–0.06)	<0.05
Site of relapse				
Bone marrow	1.00	Reference	1.00	Reference
Isolated CNS	/	/	/	/
Bone marrow + CNS	1.05 (0.5–2.2)	0.9	2.27 (0.6–8.4)	0.22
Chemotherapy/HSCT				
Chemotherapy + HSCT	1.00	Reference	1.00	Reference
Chemotherapy	1.01 (0.5–2.02)	0.982	1.54 (0.64–3.74)	0.34

Abbreviations: CNS—central nervous system, HSCT—hematopoietic stem cell transplantation.

**Table 5:** Overall survival and relapse rates stratified by treatment protocol.

	Number of Patients (%)	5-Year OS (%)	Number of Relapsed Patients	Percentage of Relapsed Patients
Interfant 99/Interfant 06	8 (2.6%)	50.0%	4	50.0%
ALL-BFM 95	54 (17.9%)	81.5%	9	16.7%
ALL IC-BFM 2002	134 (44.3%)	85.8%	17	12.7%
ALL IC-BFM 2009	103 (34.1%)	83.5%	10	9.7%
EsPhALL2004/EsPhALL2010 2010	3 (1.0%)	66.7%	1	33.3%

## Data Availability

The data presented in this study are available upon request from the corresponding author due to privacy reasons.

## Abbreviations

The following abbreviations are used in this manuscript:

ALL	Acute lymphoblastic leukemia
OS	Overall survival
HSCT	Hematopoietic stem cell transplantation
CI	Confidence interval
EFS	Event-free survival
IFI	Invasive fungal infection
